# The Influence of the Omicron Variant on RNA Extraction and RT-qPCR Detection of SARS-CoV-2 in a Laboratory in Brazil

**DOI:** 10.3390/v15081690

**Published:** 2023-08-04

**Authors:** Lívia Mara Silva, Lorena Rodrigues Riani, Juliana Brovini Leite, Jessica Mara de Assis Chagas, Laura Silva Fernandes, Romário Costa Fochat, Carmen Gomide Pinto Perches, Thiago César Nascimento, Lauren Hubert Jaeger, Marcelo Silva Silvério, Olavo dos Santos Pereira-Júnior, Frederico Pittella

**Affiliations:** 1 Faculdade de Farmácia, Universidade Federal de Juiz de Fora, Rua José Lourenço Kelmer, s/n–Campus Universitário, Juiz de Fora 36036-900, MG, Brazil; liviamarasilva@gmail.com (L.M.S.); lorena.riani@ufjf.br (L.R.R.); julianabrovini@hotmail.com (J.B.L.); jessicachaagas@gmail.com (J.M.d.A.C.); fernandes_ls@ufjf.br (L.S.F.); romariofochat@gmail.com (R.C.F.); laurenhj@hotmail.com (L.H.J.); marcelo.silverio@ufjf.br (M.S.S.); olavo.pereira@farmacia.ufjf.br (O.d.S.P.-J.); 2 Hospital Universitário, Universidade Federal de Juiz de Fora, Av. Eugênio do Nascimento, s/n, Juiz de Fora 36038-330, MG, Brazil; cpgomidep@gmail.com; 3 Faculdade de Enfermagem, Universidade Federal de Juiz de Fora, Rua José Lourenço Kelmer, s/n–Campus Universitário, Juiz de Fora 36036-900, MG, Brazil; thiago_ufjf1982@yahoo.com.br

**Keywords:** Omicron variant, SARS-CoV-2, RT-qPCR, diagnostics

## Abstract

The emergence of SARS-CoV-2 variants can affect their detection via RT-qPCR. The Omicron variant has a greater affinity for the upper respiratory system and causes clinical conditions similar to bronchitis, as opposed to the pneumonitis-like conditions caused by other SARS-CoV-2 variants. This characteristic increases the viscosity of clinical samples collected for diagnosis. Coinciding with the arrival of the Omicron variant, we observed a failure in control gene expression in our laboratory. In this report, we have optimized a rapid nucleic acid extraction step to restore gene expression and detect the presence of the SARS-CoV-2 virus. We reevaluated 3000 samples, compared variant types occurring in different time periods, and confirmed that the presence of the Omicron variant was responsible for changes observed in the characteristics of these clinical samples. For samples with large amounts of mucus, such as those containing the Omicron variant, a modification to the nucleic acid extraction step was sufficient to restore the quality of RT-qPCR results.

## 1. Introduction

Severe acute respiratory syndrome coronavirus 2 (SARS-CoV-2) is the virus responsible for the global pandemic declared by the World Health Organization (WHO) in 2020. The infection caused by this virus, known as COVID-19, affects the respiratory tract and can range from asymptomatic or mild to a severe form. As of June 2023, there have been over 768 million confirmed cases of COVID-19 and 6.9 million deaths worldwide [1]. The original strain of the virus identified in Wuhan was named B.1.1.28. In late 2020, new variants emerged in the UK (B.1.1.7—Alpha variant), South Africa (B.1.351—Beta variant), and India (B.1.617—Delta variant) [2]. These variants exhibited mutations in the spike (S) protein receptor binding domain, which contributed to increased transmissibility, with estimates ranging from 40% to 70% [3]. Another variant, the Gamma variant (also known as the P.1 variant), was identified in Brazil, and showed 17 mutations, including changes in the spike (S) protein [4].

In November 2021, a new variant with numerous mutations was detected in South Africa. This variant was named the Omicron variant (B.1.1.529) and was classified as a variant of concern (VOC) by the WHO [5]. The Omicron variant has more than 50 mutations, with 32 amino acid changes in the spike (S) protein [6]. Currently, this variant and its subvariants are dominant in several countries due to their high transmissibility. Protein S consists of two subunits, an N-terminal S1 of about 700 amino acids and a C-terminal S2 of ~600 amino acids. The spike protein plays a crucial role in viral attachment, fusion, and entry into host cells by interacting with the human angiotensin-converting enzyme receptor 2 (ACE2) [7]. Therefore, variants with multiple mutations in this protein, such as Omicron and its subvariants, require attention, as they can affect viral transmissibility, virulence, the reinfection rate, diagnostics procedures, and the effectivity of vaccines.

The heavily mutated Omicron variant has raised concerns about its detection using the golden standard RT-qPCR assays. In fact, mutations in viral RNA sequences could potentially hinder the binding of primers/probes to a specific sequence. However, it appears that the sequences developed for COVID-19 by the US Centers for Disease Control and Prevention (CDC), such as the 2019-nCoV_N1 and N2 tests, have not been significantly affected in their detection ability [8]. The US Food and Drug Administration (FDA) is actively monitoring molecular tests that may be impacted by mutations [9]. Nevertheless, it is still important to carefully examine the sequences to avoid test failure [10].

Regarding antigen tests, there is a reduced sensitivity to detecting the Omicron variant among commercially available kits [11]. The FDA and other authors have already identified diagnostic tests that contain a target gene with significantly reduced sensitivity for the Omicron variant [9,12].

In addition to variants, other crucial factors, such as the specific characteristics of clinical samples, can significantly influence the RNA extraction process. A comparative analysis of various RNA extraction methods and emphasis regarding the importance of making minor adjustments to commercial kits to successfully detect the SARS-CoV-2 virus have been reported elsewhere [13]. Some samples initially failed to exhibit a detection signal when commercial kits were used strictly following the manufacturers’ instructions. However, after implementing modifications to the ethanol evaporation time, elution incubation, and centrifugation steps, it became possible to detect signals from the target genes of SARS-CoV-2 and the internal control.

On January 2022, we observed an increasing number of inconclusive results due to control gene expression failure in our routine RT-qPCR diagnostics, particularly in turbid samples. The viral transport medium containing these samples appeared to be more viscous, mucous, and turbid. Coinciding with the arrival of these samples, there was a reported increase and predominance of the Omicron variant worldwide and in our region [14,15]. Initially, the high viscosity of the samples may have affected the extraction of nucleic acid, especially when using rapid extraction methods, necessitating adjustments to the protocol. To further investigate the possible relationship between control gene expression failure, viscous samples, and the Omicron variant, we reassessed samples collected before and during to the Omicron wave in our region.

## 2. Materials and Methods

### 2.1. Nucleic Acid Extraction

Currently, our laboratory employs rapid extraction methods to obtain viral nucleic acid. According to information from the manufacturers and the literature, the recommended protocol is directly diluting the sample with Pi-Lise^®^ Nucleic Acid Extraction Reagent (Pi-Biotech Genética Avançada, Juiz de Fora, MG, Brazil) (1:1 *v*/*v*) followed by thermal lysis, sample cooling, a quick spin, and RT-qPCR analysis [16]. The initial protocol consists of mixing 40 µL of the sample with 40 µL of the extraction reagent, homogenizing the mixture, heating it at 95 °C for 5 min, cooling for 2 min, and performing a quick spin; then the sample is ready for use in RT-qPCR.

However, upon reviewing the protocol, we considered a pre-dilution step of the sample with saline solution (0.9% NaCl) in a 1:2 ratio (1 volume of sample to 2 volumes of saline). Therefore, initially, 40 μL of the sample was diluted with 80 μL of saline and homogenized, resulting in the diluted sample. Subsequently, 40 μL of the diluted sample was combined with 40 μL of extraction reagent and homogenized. The mixture was then heated to 95 °C for 5 min, followed by a cooling period of 2 min, rendering it ready for use in RT-qPCR.

To assess the impact of dilution in RT-qPCR reactions, samples were diluted with 0.9% saline at a ratio of 1:1; 1:2, and 1:3 (*v*/*v*, sample: saline) before RT-qPCR analysis.

### 2.2. RT-qPCR Analysis

RT-qPCR analyses were conducted using the iTaq Universal Probes One-Step Kit (Bio-Rad Laboratories, Hercules, CA, USA) and the 2019-nCoV RUO kit (Integrated DNA Technologies) in an Applied Biosystem 7500 Real-Time PCR system (Thermo Fisher Scientific, Foster City, CA, USA). The cycling conditions consisted of reverse transcription at 50 °C for 15 min, initial denaturation at 95 °C for 3 min, followed by 40 cycles of denaturation at 95 °C for 15 s, and annealing and extension at 60 °C for 1 min. The final 20 µL reaction mixture contained 3.0 µL of an extracted sample, 0.5 μL of iScript enzyme mix, 10 μL of 2x iTaq PCR reaction mix, and a combination of primers/probes and water (for N1 and RNaseP: 0.8 µL of primers and probes and 5.7 µL of nuclease-free water; for N2: 0.47 µL of primers and probes, and 6.03 of µL of nuclease-free water). Additionally, samples were also evaluated using the Allplex™ SARS-CoV-2 (Seegene, Seoul, Republic of Korea) and Biomol OneStep/COVID-19 (Instituto de Biologia Molecular do Paraná—IBMP, Curitiba, PR, Brazil) multiplex kits. 

The assay performed using the Biomol OneStep/COVID-19 kit (IBMP, Curitiba, PR, Brazil) involved a reaction volume of 20 µL containing 3.0 µL of extracted sample, 4.0 µL of 5X buffer and enzymes, 1.0 µL of oligo mix, and 12 µL of water. The temperature cycles were performed as follows: 55 °C for 15 min for reverse transcription, followed by 95 °C for 3 min, and then 40 cycles of 95 °C for 15 s, and 55 °C for 40 s.

The assay carried out using the Allplex™ SARS-CoV-2 Kit (Seegene, Seoul, Republic of Korea) utilized a 20 µL reaction mixture containing 3.0 µL of extracted sample, 5.0 µL of enzyme mix (EM8), 5.0 µL of oligo mix (MOM), and 7.0 µL of water. The temperature cycles were performed as follows: 55 °C for 20 min for reverse transcription, followed by 95 °C for 15 min, and then 40 cycles of 95 °C for 15 s, and 58 °C for 30 s.

### 2.3. Analysis of SARS-CoV-2 Variant Using RT-qPCR

Identification of the SARS-CoV-2 variant was conducted using the Novaplex™ SARS-CoV-2 Variants I kit (Seegene, Seoul, Republic of Korea) via RT-qPCR, following the protocol provided by the manufacturer [17]. This kit enables the detection and differentiation of Alpha, Beta, Gamma, and Omicron variants of SARS-CoV-2 based on mutations in the S gene. The RT-aPCR analysis was performed using CFX-96 Touch Real-Time PCR Detection Systems (Bio-Rad). The cycle was performed at 50 °C for 20 min for reverse transcription, followed by 95 °C for 15 min, and then 3 cycles of 95 °C for 10 s, 60 °C for 40 s, and 72 °C for 20 s, and then 42 cycles of 95 °C for 10 s, 60 °C for 15 s, and 72 °C for 10 s. The assay was performed using a 20 µL reaction mixture.

### 2.4. Ethics

This study was approved by the local research ethics committee, under the number CAAE 48021321.3.0000.5147.

## 3. Results and Discussion

**Figure 1:**
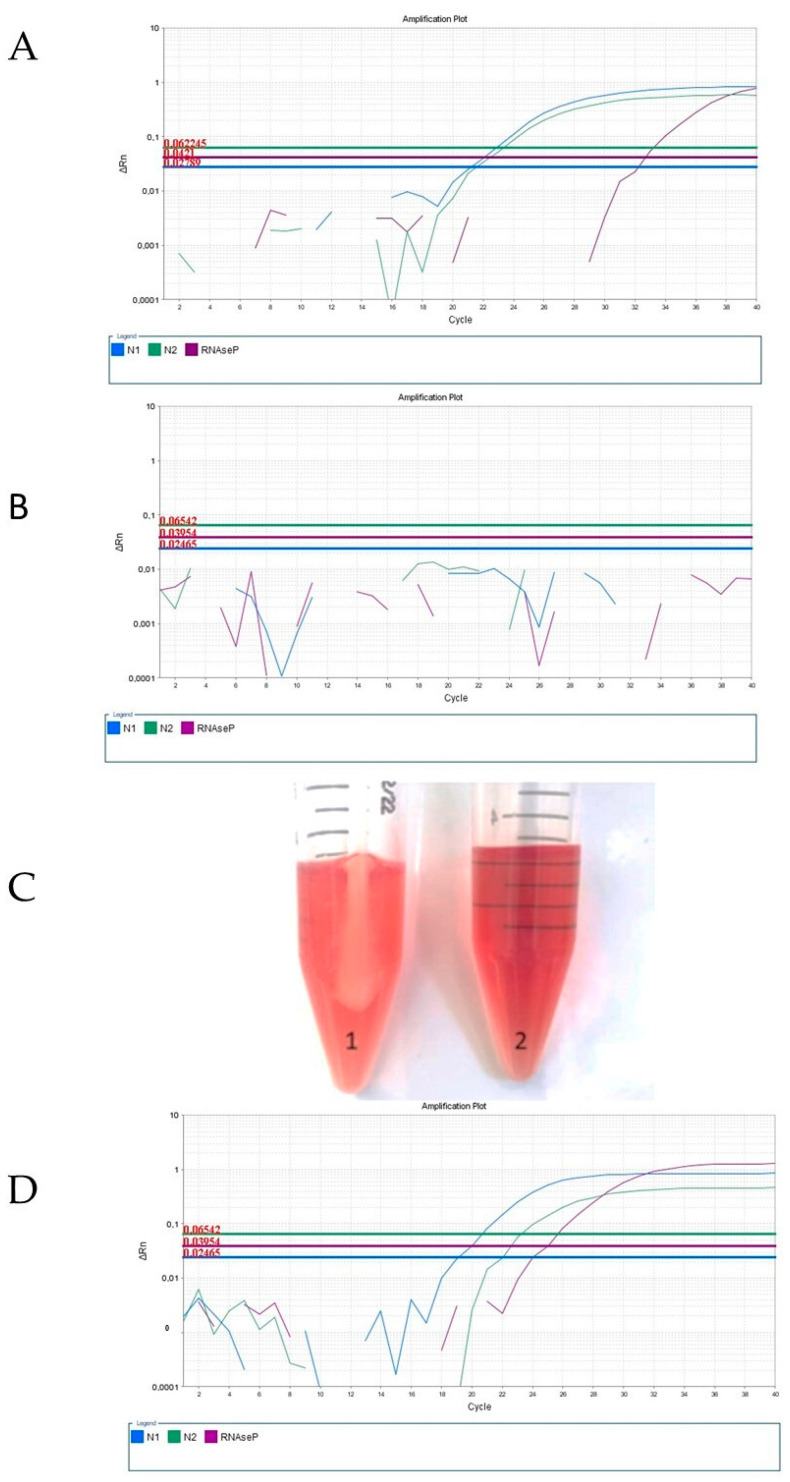
Amplification curves of different samples containing SARS-CoV-2 (via RT-qPCR) using the iTaq Universal Probes One-Step Kit and the 2019-nCoV RUO kit. (**A**) Representative amplification plot of a sample obtained in June 2021, corresponding to Alpha, Beta, Gamma, or Delta variants. (**B**) Amplification plot of an undiluted sample obtained in January 2022, probably corresponding to the Omicron variant. (**C**) Illustrative picture of nasopharyngeal swab sample that arrived in the middle of February 2022 (1) compared with control viral transport medium and (2) showing high turbidity and mucus presence in the sample. (**D**) Amplification plot of the same sample of from Figure 1B, obtained in January 2022, probably corresponding to the Omicron variant, in which a pre-dilution with saline in a 1:2 ratio (1 sample: 2 saline, *v*/*v*) was applied.

Following the original RT-qPCR protocol, we performed gene amplification diagnosis (Figure 1A) until January 2022, which corresponded to the circulation of the Alpha, Beta, Gamma, and Delta variants. However, when applying these methods to clinical samples collected from January 2022 onwards, we encountered failure in control gene amplification, leading to numerous inconclusive results (Figure 1B). Upon reviewing various parameters in our procedure, we identified the main difference to be the appearance of the samples received in our laboratory (Figure 1C).

To restore control gene amplification, we refined the sample preparation protocol for extracting genetic material using the rapid extraction reagent. Initially, we evaluated a series of dilutions before extraction (Supplementary Material Figure S1) and found that pre-diluting the sample with saline was sufficient to restore gene amplification (fluorescence signals) and improve the quality of the RT-qPCR analysis (Figure 1D). Rapid nucleic acid extraction methods provide non-purified nucleic acids, which are suitable for analysis using hydrolysis probe-based assays (i.e., Taqman technology). However, this material is susceptible to degradation by nucleases if non-protected. Sputum and mucus contain substances that can degrade nucleic acids and/or inactivate proteins responsible for the lysis or preservation of the genetic material [18]. One important group of proteins found in mucus is mucins, a family of glycoproteins with various properties, including surfactants functions [19]. Additionally, the presence of excessive inflammatory cell residues (lipids, carbohydrates, proteins) may interfere with the polymerase chain reaction, leading to impaired gene amplification.

Substances that are co-extracted can interfere with or hinder PCR reactions, leading to partial or complete inhibition, which may increase the likelihood of false negatives. Regarding DNA quantification, even a minor inhibition of a qPCR reaction can slow down the reaction kinetics, leading to an underestimation of DNA concentration or even a failure to detect it. Some previous studies have employed sample dilution of up to 10 times in water to mitigate the inhibition of PCR assays [20,21].

**Figure 2:**
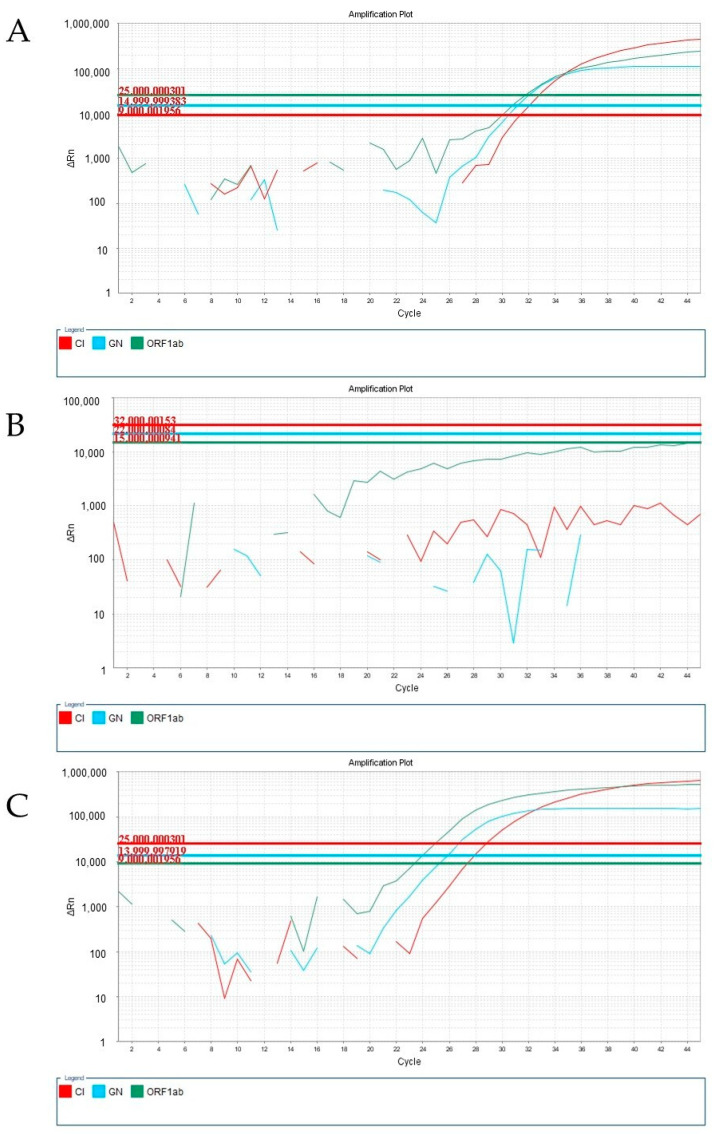
Amplifications of different samples containing SARS-CoV-2 (via RT-qPCR) analyzed using the Biomol OneStep COVID-19 (IBMP) Kit. (**A**) Amplification curve of the sample obtained in June 2021, corresponding to the Alpha, Beta, Gamma, or Delta variants. (**B**) Amplification curve of the undiluted sample, obtained in January 2022, probably corresponding to the Omicron variant. (**C**) Amplification curve of the same sample as in B, to which a pre-dilution with saline at a 1:2 ratio (1 sample: 2 saline) was applied.

**Figure 3:**
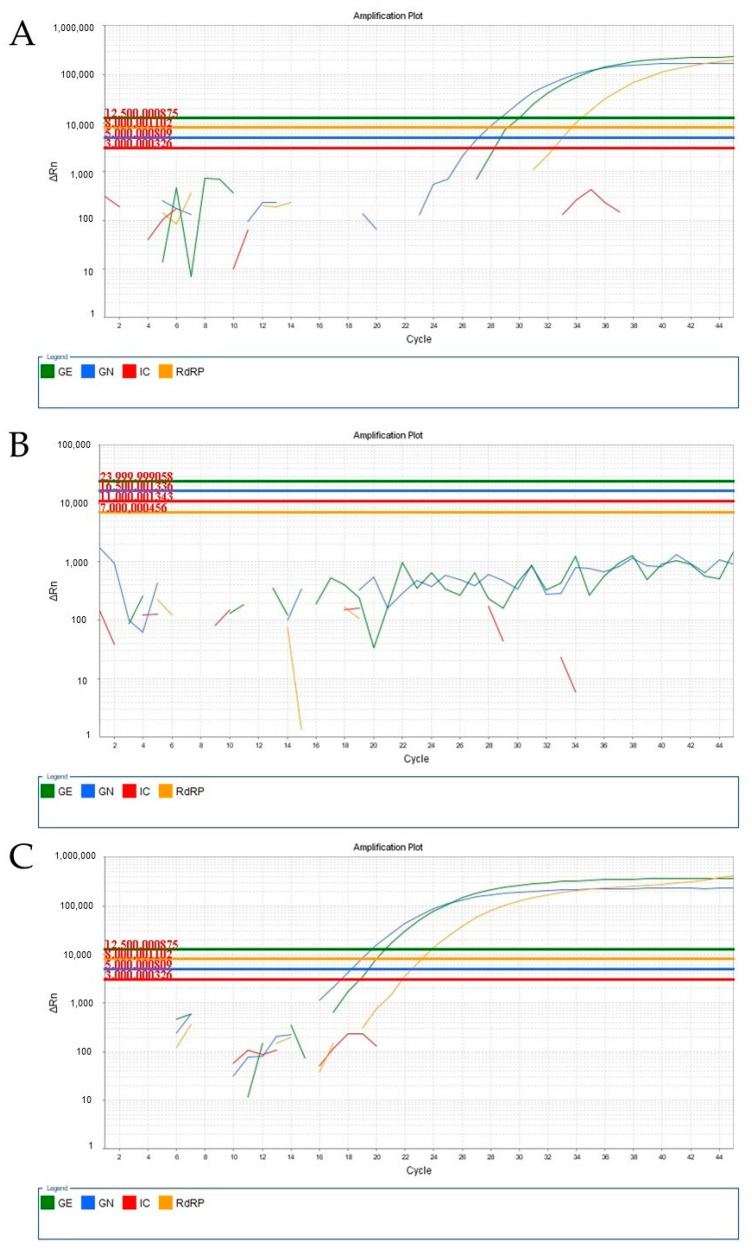
Amplifications of different samples containing SARS-CoV-2 (via RT-qPCR) analyzed using the Allplex SARS-CoV-2 (Seegene) kit. (**A**) Amplification curve of the sample obtained in June 2021, corresponding to the Alpha, Beta, Gamma, or Delta variants. (**B**) Amplification curve of the undiluted sample, obtained in January 2022, probably corresponding to the Omicron variant. (**C**) Amplification curve of the same sample as in B, to which a pre-dilution with saline at a 1:2 ratio (1 sample: 2 saline) was applied.

We also investigated the impact of the viscous samples (probably containing the Omicron variant) using different RT-qPCR kits. When using the Biomol OneStep COVID-19 (IBMP, Curitiba, PR, Brazil) Kit and the Allplex™ SARS-CoV-2 (Seegene, Seoul, Republic of Korea) kit, we observed a consistent amplification pattern. In samples containing the Alpha, Beta, Gamma, or Delta variants, we could visualize the amplification corresponding to SARS-CoV-2 targets (Figure 2A and Figure 3A). However, for undiluted samples obtained in January 2022, no amplification curve was observed (Figure 2B and Figure 3B). Interestingly, amplification was restored when the same sample was diluted with saline at a ratio of 1:2 (Figure 2C and Figure 3C).

**Figure 4:**
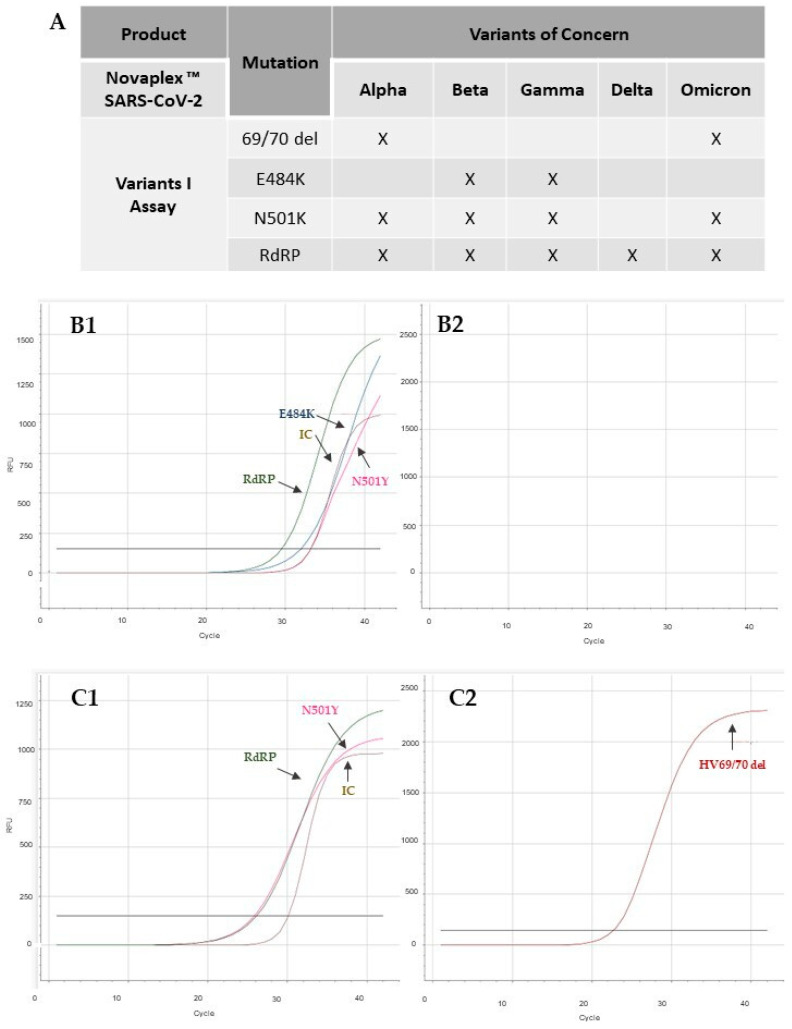
Analysis and differentiation of SARS-CoV-2 variants. (**A**) Detection and differentiation of mutations according to the Novaplex™ SARS-CoV-2 Variants I Assay kit. (**B**) Representative result of a sample from September 2021, with amplification of the E484K gene and others, indicating that it is the Beta or Gamma variant sample (**B1**), and without amplification of the HV69/70 del mutation gene (**B2**), indicating that it is not the Omicron variant. (**C**) Representative result of a sample from January 2022, with amplification of the N5011Y and 69/70 del mutation genes (**C1**,**C2**), indicating that it is the Omicron variant.

To confirm the association between the viscous samples exhibiting control gene expression failure (inconclusive results) and the presence of the Omicron variant, 50 samples were selected from the periods of August 2021 (pre-Omicron) and January 2022 (peak of Omicron) and analyzed using the Novaplex™ SARS-CoV-2 Variants I kit. This kit enables the differentiation of variants based on the detection of specific mutations, including E484K, N501Y, and HV69/70 del mutations, in addition to the RdRp gene, which is common to all variants (Figure 4A). The E484K mutation, found in the Beta and Gamma variants, is associated with increased binding affinity to ACE receptors. The N501Y mutation is present in the Alpha, Beta, Gamma, and Omicron variants and also affects the ACE receptors’ affinity. The HV69/70 deletion mutation is found in the Alpha and Omicron variants [17,22].

All samples collected from August to December 2021 exhibited the presence of different genes (Figure 4B1), indicating that they corresponded to the Beta, Gamma, or other variants not identified by the kit, such as Delta. However, these samples did not show amplification for the HV69/70 del mutation (Figure 4B2), suggesting that they did not correspond to the Omicron variant. This finding was consistent with the prevalence of other variants in our region [14,15]. In some samples, we observed signal amplification of RdRP and the absence of amplification of targets E484K, N501Y, and HV69/70 del, which are not present in the Delta variant. Given that the pre-Omicron period was characterized by a wave of the Delta variant, it is possible that these samples corresponded to this variant. However, all samples collected in January 2022, which initially yielded inconclusive results in the tests before the dilution process, exhibited amplification for the HV69/70 del and N501Y mutation, confirming that they indeed corresponded to the Omicron variant (Figure 4C1,C2). The individual results of these analyses are shown in Table S1 (Supplementary Material). The results obtained in this analysis indicate that although the previous dilution of the sample allowed identification of the Omicron variant, the adjustment in the extraction protocol did not interfere with the detection of other variants.

Recent studies indicate that Omicron replicates more readily in the upper airways than in the lungs [23], suggesting an important change in clinical symptoms of COVID-19 with the Omicron variant. Symptoms caused by the Omicron variant are more restricted to the upper respiratory system and produce clinical conditions such as bronchitis, nasal discharge, sore throat, and sneezing, while the previous variants tend to result in pneumonitis-like conditions [23,24]. In fact, a strong feature of bronchitis is the production of sputum and viscous secretion [25], which is well-correlated with the characteristics of the viscous clinical samples arriving in our laboratory (Figure 1C).

**Figure 5:**
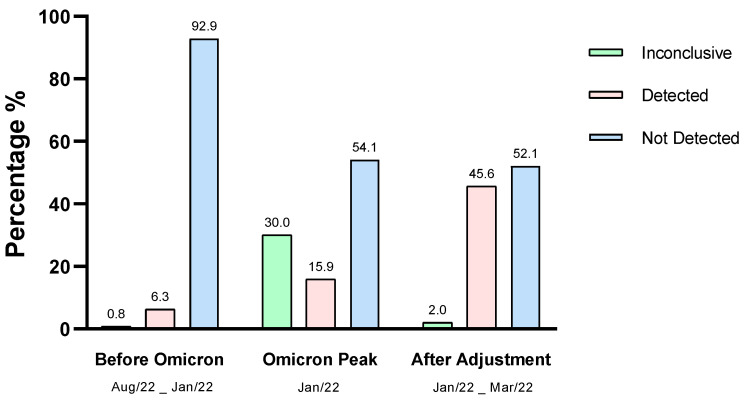
Results of RT-qPCR analyses of samples obtained from 21 August to 22 March. The results show the difference between inconclusive, not detected, and detected results in the periods before the appearance of the Omicron variant, at the peak of Omicron, and after adjustment of the nucleic acid extraction protocol.

Further, we conducted a reassessment of three thousand (3000) diagnostic test results from August 2021 to March 2022 to confirm the impact of the emergence of the Omicron variant on RT-qPCR analysis and the effect of pre-dilution of the biological sample. The tests covered three periods: pre-Omicron variant, peak prevalence of the Omicron variant without sample pre-dilution, and after adjustment of the protocol for the Omicron variant. In the period before the emergence of the Omicron variant, the rate of inconclusive results was 0.83%. However, after the appearance of the Omicron variant in January 2022, this rate significantly increased to 30.0%. Nonetheless, after adjusting the protocol for nucleic acid extraction and re-establishing control gene amplification, the inconclusive results rate dropped to 2.0%. This indicates that pre-dilution of the biological sample can decrease interferences in the RT-qPCR assay, resulting in a significant reduction in inconclusive results (Figure 5).

## 4. Conclusions

Indeed, changes in sample characteristics and the emergence of new variants necessitate a critical analysis for the adjustment of established RT-qPCR conditions. This includes identifying specific mutations, examining the annealing regions of primers and probes, and considering the overall relationship between clinical effects and the quality of biological samples received in diagnostic laboratories. As observed in this study, samples collected from patients infected with the Omicron variant of SARS-CoV-2 exhibited characteristics such as viscosity and turbidity, which affected the expression of the control gene in our laboratory. The adjustment of sample preparation through pre-dilution before extraction proved effective in restoring the accuracy of the analysis. This appoach may be extended to other reagents and methods, and highlights the importance of adapting protocols to account for changes in sample characteristics and emerging variants.

## Data Availability

The data presented in this study are available in Supplementary Material.

## Supplementary Materials

The following supporting information can be downloaded at: https://www.mdpi.com/article/10.3390/v15081690/s1, Figure S1: Amplification of positive sample containing SARS-CoV-2 by RT-qPCR, from samples collected in January 2022 and analyzed using Allplex kit; Table S1: Results obtained in the analysis of SARS-CoV-2 variants.
