# Supplementary material for: The Influence of the Omicron Variant on RNA Extraction and RT-qPCR Detection of SARS-CoV-2 in a Laboratory in Brazil
Source: Viruses. 2023 Aug 4;15(8):1690. doi: 10.3390/v15081690 (PMC10458054; doi:10.3390/v15081690)
Supplement: Supplementary file 1 [file viruses-15-01690-s001.zip › viruses-2503522-supplementary.pdf]

viruses

Supplementary Material

The Influence of the Omicron Variant on RNA Extraction and RT-qPCR Detection of SARS-CoV-2 in a Laboratory in Brazil

Lívia Mara Silva [1], Lorena Rodrigues Riani [1], Juliana Brovini Leite [1], Jessica Mara de Assis Chagas [1], Laura Silva Fernandes [1], Romário Costa Fochat [1], Carmen Gomide Pinto Perches [2], Thiago César Nascimento [3], Lauren Hubert Jaeger [1], Marcelo Silva Silvério [1], Olavo dos Santos Pereira-Júnior [1] and Frederico Pittella [1,*]

[1] Faculdade de Farmácia, Universidade Federal de Juiz de Fora, Rua José Lourenço Kelmer, s/n–Campus Universitário, Juiz de Fora 36036-900, MG, Brazil
[2] Hospital Universitário, Universidade Federal de Juiz de Fora, Av. Eugênio do Nascimento, s/n, Juiz de Fora 36038-330, MG, Brazil
[3] Faculdade de Enfermagem, Universidade Federal de Juiz de Fora, Rua José Lourenço Kelmer, s/n–Campus Universitário, Juiz de Fora 36036-900, MG, Brazil
* Correspondence: frederico.pittella@ufjf.br; Tel.: +55-32-2102-3803

Results

Initially, we evaluated the optimal dilution ratio to improve amplification and achieve better results in the sample's RT-qPCR assay. By using a pre-dilution of viscous samples, we successfully restored the amplification of the control gene. The results indicated no significant difference between the dilutions (1:1; 1:2 and 1:3) for the detection of positive samples, suggesting that any dilution is effective and provides better results compared to analyzing the sample without dilution (Fig. S1A-S1D). The dilution recommended was the ratio 1:2, as it minimizes the residual interference of PCR and fluorescence inhibitors, resulting in a better amplification signal.

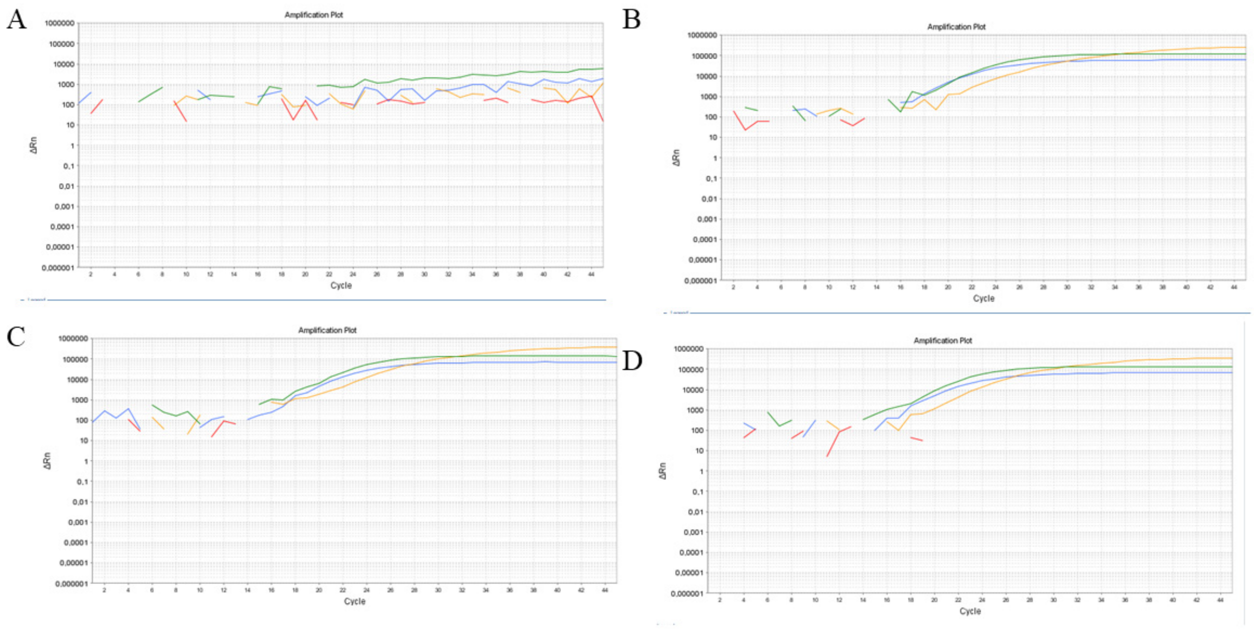

Figure S1. Amplification of positive sample containing SARS-CoV-2 by RT-qPCR, from samples collected in January 2022 and analyzed using Allplex kit. A) Amplification of the sample without dilution. B) Sample amplification after 1:1 dilution. C) Sample amplification after 1:2 dilution (1 sample: 2 saline). D) Sample amplification after 1:3 dilution (1 sample: 3 saline).

All samples collected from August to December 2021 exhibited the presence of different genes, indicating that they corresponded to the Beta, Gamma or other variants not identified by the kit, such as Delta. However, all samples collected in January 2022, which initially yielded inconclusive results in the tests before the dilution process, exhibited amplification for the HV69/70del and N501Y mutation, confirming that they indeed corresponded to the Omicron variant. The individual results of probable variants, as well as the CT of the analyzed targets, are presented in Table S1.

Table S1. Results obtained in the analysis of SARS-Cov-2 variants.

Period	Sample ID	Genes (CT)				Probable variant
		E484K	RdRP	N501Y	HV69/70 del	
Pre-Omicron	1	-	22.93	-	-	Delta
	2	27.18	20.95	26.32	-	Beta or Gamma
	3	30.52	25.98	31.48	-	Beta or Gamma
	4	31.94	29.52	33.23	-	Beta or Gamma
	5	31.97	28.74	28.54	-	Beta or Gamma
	6	-	26.94	-	-	Delta
	7	-	27.85	-	-	Delta
	8	-	27.45	-	-	Delta
	9	-	21.59	-	-	Delta
	10	-	19.61	-	-	Delta
	11	-	24.06	-	-	Delta
	12	-	27.30	-	-	Delta
	13	-	22.47	-	-	Delta
	14	-	36.22	-	-	Delta
	15	-	32.57	-	-	Delta
	16	-	31.31	-	-	Delta
	17	29.80	31.50	30.27	-	Beta or Gamma
	18	-	25.95	-	-	Delta
	19	-	30.71	-	-	Delta
	20	-	23.60	-	-	Delta
	21	-	23.45	-	-	Delta
	22	-	20.61	-	-	Delta
	23	39.40	39.28	31.57	-	Beta or Gamma
	24	-	22.77	-	-	Delta
	25	-	29,08	-	-	Delta
Omicron Peak	26	-	37.73	31.85	30.64	Omicron
	27	-	39.11	35.57	32.58	Omicron
	28	-	37.22	33.96	30.49	Omicron
	29	-	25.49	27.13	23.61	Omicron
	30	-	22.13	22.60	19.02	Omicron
	31	-	25.98	26.92	23.16	Omicron

	32	-	26.26	25.64	23.48	Omicron
	33	-	29.83	28.28	25.75	Omicron
	34	-	27.39	26.72	23.87	Omicron
	35	-	28.74	26.76	26.81	Omicron
	36	-	24.15	24.69	21.49	Omicron
	37	-	30.63	29.50	27.10	Omicron
	38	-	26.12	25.82	22.76	Omicron
	39	-	29.15	29.07	25.99	Omicron
	40	-	32.65	30.58	27.78	Omicron
	41	-	25.41	23.89	24.06	Omicron
	42	-	27.00	27.90	25.02	Omicron
	43	-	36.81	35.00	31.70	Omicron
	44	-	28.00	26.54	23.99	Omicron
	45	-	25.29	25.39	22.36	Omicron
	46	-	31.74	29.05	33.78	Omicron
	47	-	27.42	28.92	26.36	Omicron
	48	-	22.77	23.93	20.46	Omicron
	49	-	35.53	34.78	31.52	Omicron
	50	-	26.53	28.23	25.17	Omicron
